# Nitrogenase FeMoco investigated by spatially resolved anomalous dispersion refinement

**DOI:** 10.1038/ncomms10902

**Published:** 2016-03-14

**Authors:** Thomas Spatzal, Julia Schlesier, Eva-Maria Burger, Daniel Sippel, Limei Zhang, Susana L.A. Andrade, Douglas C. Rees, Oliver Einsle

**Affiliations:** 1 Institute for Biochemistry, Albert-Ludwigs-Universität Freiburg, Albertstrasse 21, 79104 Freiburg, Germany; 2 Howard Hughes Medical Institute, Division of Chemistry and Chemical Engineering, California Institute of Technology, Pasadena, California 91125, USA; 3 Division of Chemistry and Chemical Engineering, California Institute of Technology, Pasadena, California 91125, USA; 4 BIOSS Centre for Biological Signalling Studies, Albert-Ludwigs-Universität Freiburg, Hebelstrasse 25, 79104 Freiburg, Germany

## Abstract

The [Mo:7Fe:9S:C] iron-molybdenum cofactor (FeMoco) of nitrogenase is the largest known metal cluster and catalyses the 6-electron reduction of dinitrogen to ammonium in biological nitrogen fixation. Only recently its atomic structure was clarified, while its reactivity and electronic structure remain under debate. Here we show that for its resting *S*=3/2 state the common iron oxidation state assignments must be reconsidered. By a spatially resolved refinement of the anomalous scattering contributions of the 7 Fe atoms of FeMoco, we conclude that three irons (Fe1/3/7) are more reduced than the other four (Fe2/4/5/6). Our data are in agreement with the recently revised oxidation state assignment for the molybdenum ion, providing the first spatially resolved picture of the resting-state electron distribution within FeMoco. This might provide the long-sought experimental basis for a generally accepted theoretical description of the cluster that is in line with available spectroscopic and functional data.

## Abstract (web summary)

The [Mo:7Fe:9S:C] iron-molybdenum cofactor (FeMoco) of nitrogenase is a large metal cluster with an important role in biological nitrogen fixation. Here, the authors use spatially resolved refinement of the anomalous scattering contributions of the iron atoms to determine the resting-state electron distribution of FeMoco.

## Graphical abstract

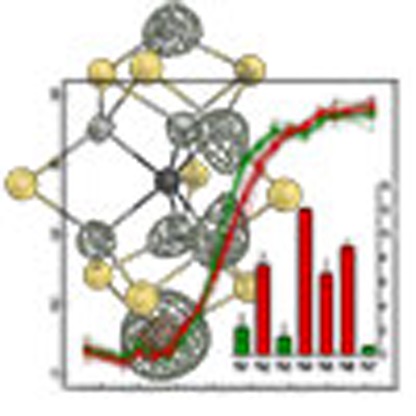

Biological nitrogen fixation is an essential process required to tap into the predominant reservoir of bioavailable nitrogen, atmospheric N_2_. Although at any given time an estimated 99% of all nitrogen cycling the biosphere are present in this form^1^, only a single enzymatic reaction has evolved to break the stable triple bond of the dinitrogen molecule. The enzyme nitrogenase is a complex two-component system consisting of the heterotetrameric MoFe protein (NifD_2_K_2_), where substrates are reduced, and the dimeric Fe protein (NifH_2_) that serves as the only known electron donor for N_2_ reduction and as the site of ATP hydrolysis^2^,^3^. Nitrogenase reduces N_2_ according to equation (1), as well as a series of alternative substrates, prominently including carbon monoxide that is converted to a mixture of unsaturated hydrocarbons with potential relevance for biofuel production^4^,^5^.


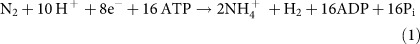


The MoFe protein of nitrogenase contains two unique metal clusters, the [8Fe:7S] P-cluster, an electron transfer centre with an unusual 8Fe^+2^ ground state as isolated, and FeMo cofactor (FeMoco), the site of substrate reduction. FeMoco was originally described as a [Mo:7Fe:9S]:*R*-homocitrate entity with a central cavity surrounded by six coordinatively unsaturated iron ions^6^,^7^. The arrangement intuitively suggested a coordination site for substrates within the central cavity, but this hypothesis was challenged by the structural inertness of the cluster. We then found that a central light atom that was masked by an unfavourable occurrence of Fourier series termination artefacts was eventually located in the cofactor centre^8^. Only recently this light atom was identified as a carbon species^9^,^10^ that originates from *S*-adenosylmethionine^11^ and does not exchange during catalysis^12^. It thus constitutes a stabilizing element that provides rigidity to the cluster ground state and explains the observed structural homogeneity. Nitrogenase MoFe protein is commonly isolated in a reduced form, with a diamagnetic, all-ferrous P-cluster (P^N^) and the FeMo cofactor in an *S*=3/2 state (FeMoco^N^) that has been extensively characterized by electron paramagnetic resonance (EPR) and enhanced nuclear double-resonance (ENDOR), X-ray absorption spectroscopy (XAS) and Mössbauer spectroscopies^13^,^14^,^15^,^16^,^17^. A recent analysis of single-crystal EPR data for the FeMoco^N^ state revealed the orientation of the magnetic **g**-tensor of the cluster within MoFe protein, highlighting that the electrostatic potential field induced by the protein matrix orients the spin system^18^. In this study, the *g*_z_ main axis of the magnetic tensor oriented along the intrinsic threefold symmetry axis of FeMo cofactor, whereas the *g*_y_ axis was found to align with a cluster edge formed by atoms Fe1, Fe3, Fe7 and Mo (Fig. 1a).

The final clarification of the structure of FeMo cofactor, however, did not help to answer key mechanistic question, and the exact binding site for substrates as well as the mechanism of their reduction remain to be elucidated (Table 1). A deeper understanding of complex bioinorganic systems commonly arises only from a combination of methodologies, with structural information complemented by spectroscopy, but also by theoretical calculations and synthetic models. Small-molecule compounds able to activate dinitrogen were known for decades^19^, but only more recent compounds, such as [HIPTN_3_N]Mo(N_2_) by Schrock^20^ and [Mo(N_2_)_2_(PNP)]_2_(μ-N_2_) by Nishibayashi^21^, achieved several cycles of catalytic turnover and were based on molybdenum as the reactive species. More recently, Hou and co-workers presented a catalytic titanium hydride complex, [(C_5_Me_4_SiMe_3_)Ti]_4_(μ^3^-NH)_2_(μ^2^-H)_4_^22^. Peters *et al*. synthesized a first iron compound [(TBP)Fe(N_2_)] that achieved catalysis^23^, and Holland and co-workers most recently extended this to a Fe-based system with iron and carbon ligands that is chemically reminiscent of FeMoco^24^. Each of these studies represented a major advance in synthetic inorganic chemistry and the catalysis of dinitrogen reduction, but they did not provide unambiguous evidence for the mode of action of the enzyme. Because of the inertness of the resting state, the investigation of inhibitor/substrate interactions with FeMo cofactor was limited to spectroscopic methods, and here an incomplete understanding of the electronic structure of FeMoco precluded the precise assignment of signals^19^. Very recently, the discovery of CO-bound FeMoco by X-ray crystallography provided structural information on a functionalized state of the active site for the first time^25^. The utility of such structural data for guiding further spectroscopic and theoretical studies is obvious and may help to advance understanding of the mechanism of nitrogenase substantially. However, a prerequisite for this approach is a detailed electronic description of FeMoco, and in spite of numerous studies by many of the leaders is their field, theoretical approaches to date have not succeeded to produce a generally accepted model^19^. Different models proposed ligand binding to the central cavity^26^,^27^, but also to various positions on the cluster surface^28^,^29^, and most recently the CO adduct inspired a proposal where N_2_ is activated in the exact same position, made possible through concomitant H_2_ evolution^30^.

Experimental data on the actual electron distribution within FeMoco are scarce, largely because spectroscopic techniques can hardly resolve and characterize individual Fe ions in among the 30 Fe sites per MoFe protein. On the other hand, the precise spatial resolution of experimental data is a hallmark of diffraction techniques, but although X-ray crystallography was highly instrumental in describing the structure of the cluster, it is not straightforward to use as a tool for investigating oxidation states of individual metals. We have recently presented a method to address this problem and extract further information about the electronic structure of every single metal site from X-ray diffraction data^31^. This strategy exploits the property of anomalous scattering that describes the breakdown of Friedel's law^32^, the intrinsic inversion symmetry of diffraction (*F*(**S**)=*F*(–**S**)), in the proximity of an X-ray absorption edge. Anomalous scattering is routinely used to solve the crystallographic phase problem for the *ab initio* structure determination of proteins^33^, and its magnitude across an edge is proportional to the absorption of X-rays. With diffraction data sets collected at various X-ray energies, it is possible to refine the anomalous scattering contributions *f* ″ and *f* ′ individually for each anomalous scatterer in a structure (Fig. 1b and Supplementary Table 1). The relevance of such a spatially resolved anomalous dispersion (SpReAD) analysis arises from the fact that the absorption properties of a given scatterer reflect the chemical environment, but also the electronic state of the atom. Upon oxidation, the ionization energy of the inner-shell electrons that determines the position of an absorption edge will increase, making the edge position a strong indicator for oxidation state and enabling us to carry out this analysis at any given point in space, that is, for each single atom of a given type. The method was successfully applied to identify the localized electron in a reduced [2Fe:2S] cluster^31^, and to characterize an additional Fe site recently discovered in MoFe protein^34^.

Here we have applied the SpReAD methodology to the enzyme nitrogenase as one of the largest and most complex metalloproteins known to date. We show that the edge positions, and thus most likely the oxidation states of the individual iron sites are distinct and suggest an electron distribution that matches up with the Mo(III) ion present in the cluster to yield the observed *S*=3/2 state. Understanding the electronic structure of FeMoco is an essential prerequisite for a concise functional analysis of this unique centre, paving the way for possible applications in catalysis and bioengineering.

## Results

### The SpReAD experiment

**Figure 1:**
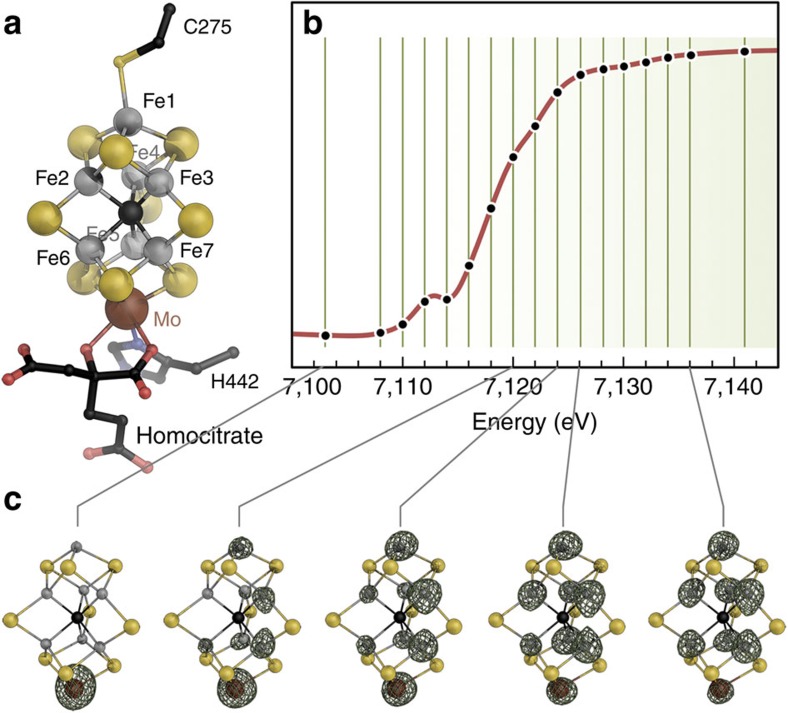
FeMo cofactor, the active site cluster of nitrogenase. (**a**) Structure of FeMo cofactor as observed in MoFe protein from *A. vinelandii* (PDB-ID 3U7Q). The structure represents the *S*=3/2 FeMoco^N^ state, a stable resting state that does not bind substrates. (**b**) Strategy for spatially resolved anomalous dispersion (SpReAD) refinement. A XAS spectrum or a fluorescence scan of the iron K-edge (red) is chosen to determine energies for the collection of full diffraction data sets. In these, the anomalous scattering contribution can be refined for individual atoms. (**c**) Anomalous difference electron density maps contoured at the 3*σ* level around FeMo cofactor, calculated for data sets taken at the indicated positions along the iron K-edge. The magnitude of the electron density peak does not directly reflect *f*″, but the increase of signal is clearly visible. Note that strong features appear first for the most electron-rich atoms, Fe1, Fe3 and Fe7 (orientation as in **a**).

**Figure 2:**
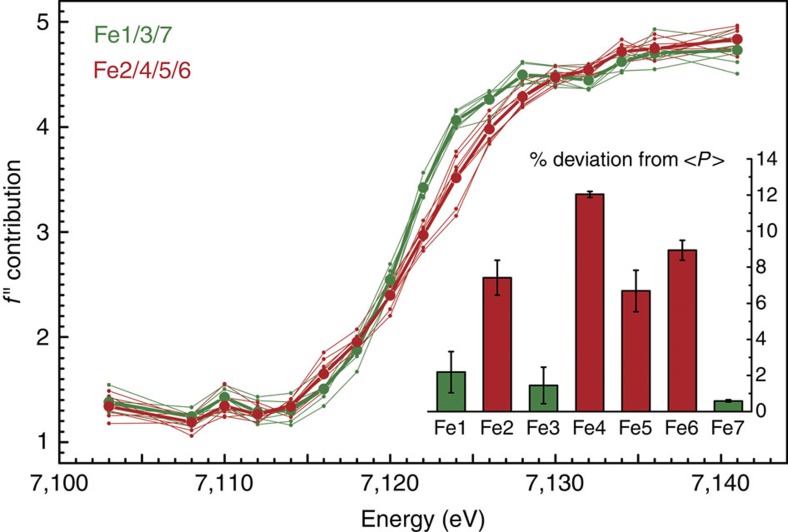
Refined scattering curves for the Fe atoms of Nitrogenase FeMo cofactor. Individual SpReAD profiles for the 14 Fe atoms in the two copies of FeMoco in the asymmetric unit of the *P*2_1_ unit cell group into two populations, with Fe1, Fe3 and Fe7 showing lower edge energies than the remaining metal ions. The average of each of the populations is plotted in bold lines. The inset shows the deviation from the average SpReAD profile for the Fe ions in the P clusters (<P>), calculated as a relative change of area under the rising edge portion of the individual profiles. In the P^N^ state, all sites of P-cluster are Fe^+2^ and were thus used as internal reference (Supplementary Fig. 2). The iron nomenclature is based on PDB entry 3U7Q.

**Figure 3:**
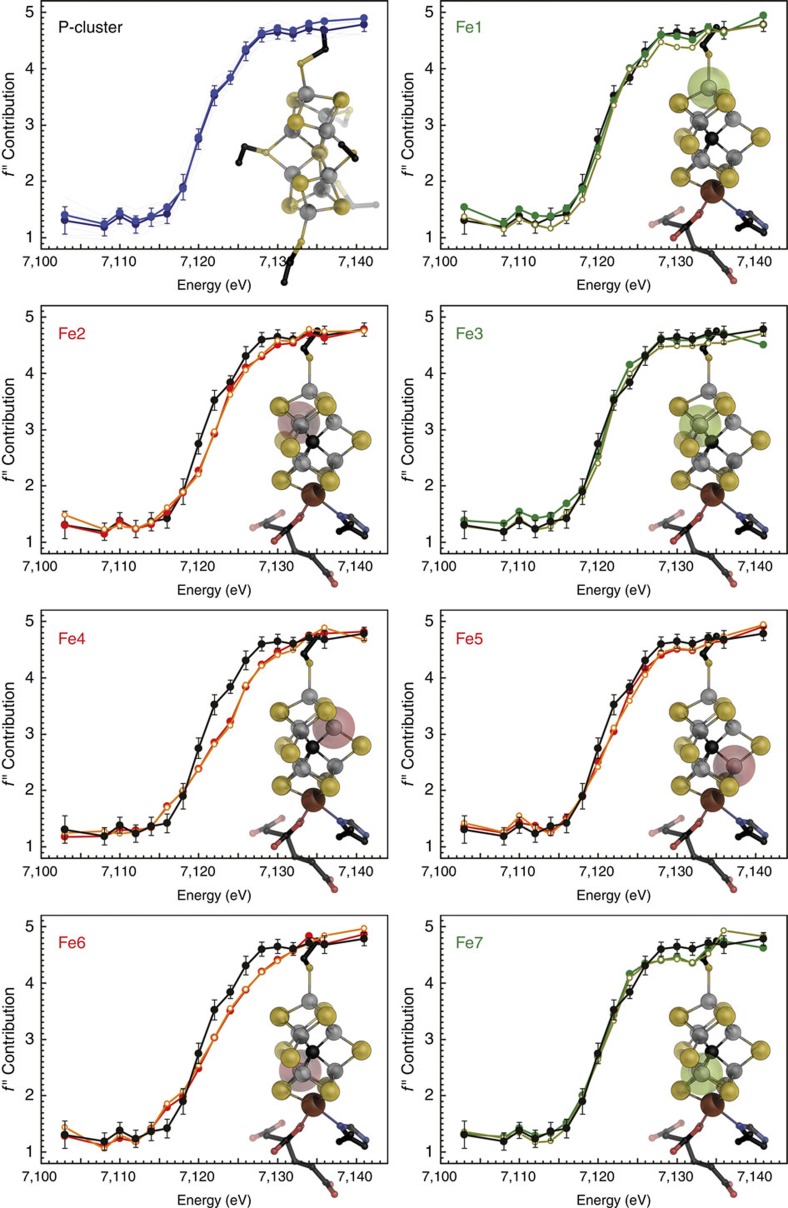
Scattering curves for the Fe atoms of nitrogenase FeMo cofactor. In the PN state of P-cluster, all irons are in the Fe(II) state and were averaged as an internal reference for ferrous iron (black). Individual SpReAD profiles for the seven iron atoms of FeMoco (shown in ball-and-stick representation) align with this standard for Fe1, Fe3 and Fe7, whereas the remaining Fe2, Fe4, Fe5 and Fe6 were shifted to higher energies, indicating a more oxidized state.

We have analysed MoFe protein in the FeMoco^N^ state using spatially resolved anomalous dispersion data collected at 17 discrete energies along the iron K-edge (Supplementary Table 1). In our anomalous difference electron density maps, the magnitude of the anomalous signal increased across the absorption edge as expected (Fig. 1c). A structural model of MoFe protein was refined against the data set collected at 7,103 eV and inspection of the electron density maps confirmed the P-cluster to be entirely in the P^N^ state, as Fe sites 5 and 6 would change their position upon oxidation of the cluster^35^. For every single iron atom in FeMo cofactor and in the P-cluster, an individual refinement of the anomalous *f* ″ and the dispersive *f* ′ contribution of anomalous scattering was carried out as described previously^31^. The asymmetric unit of the *P*2_1_ crystals of *A. vinelandii* MoFe protein contained one heterotetramer with two copies each of the P-cluster (8 Fe) and the FeMoco (7 Fe), so that the two instances of each cluster could be inspected separately for comparison and yielded virtually identical results (Figs 2 and 3).

### Anomalous dispersion refinement of individual Fe atoms

Thirty individual Fe atoms were included in the refinement, as well as the other relevant anomalous scatterers present in the crystal, Mo and S. The absorption behaviour of these elements is featureless around the iron K-edge, but they contributed to total anomalous scattering and were thus included as the sum of all atoms of the given type. Refinement of the anomalous and dispersive contributions of the iron atoms resulted in characteristic edge features (Figs 2 and 3). We also considered whether the relative orientation of the metal clusters to the synchrotron beam influences the individual absorption edges, but were unable to find any correlation, indicating that these only play a minor role in our analysis (Supplementary Fig. 1). As expected, no significant differences were observed for the 8 Fe ions of the P-cluster in the reduced P^N^ state, yielding a plot of the absorption edge that served as an internal reference for ferrous iron in near-tetrahedral coordination geometry (Fig. 3). For the FeMo cofactors, the individual iron atoms showed clear differences in the shape and position of the absorption edges, providing fingerprints of their electronic structure. The seven iron sites of each FeMo cofactor grouped into two distinct subpopulations, resulting in edge profiles that for three irons were shifted to lower energy with respect to the other four (Figs 2 and 3).

This finding was consistent in different data sets and across two different synchrotron radiation sources. In each case, the iron sites with an edge position at lower energy were Fe1, Fe3 and Fe7, whereas the profiles for Fe2, Fe4, Fe5 and Fe6 refined to higher energies (Fig. 2, inset). Although we refrain from drawing quantitative conclusions about individual iron oxidation states based on single edges at the present state of the method, we can use the all-ferrous P^N^-cluster as a defined, internal standard for a reference analysis. As in X-ray absorption spectroscopy, the ligand environment has an influence on the shape and position of the obtained edge. In FeMo cofactor, Fe1 has a largely symmetric ligand field similar to the irons of P^N^ (Fig. 3a–c). The remaining Fe2 to Fe7 form the corners of a trigonal prism, each having an identical ligand field, a slightly distorted tetrahedron with one sulfido ligand exchanged by the central carbide (Fig. 1c and Supplementary Fig. 2). Nevertheless, the SpReAD profiles for Fe1, Fe3 and Fe7 align almost perfectly with the average P^N^ irons, indicating a ferrous state. When calculating edge shifts as deviations from the average SpReAD profile of the P-clusters (Fig. 2, inset), the same tendencies are obvious, with minimal deviations for Fe1, Fe3 and Fe7. This further implies that Fe2, Fe4, Fe5 and Fe6 are present in a more oxidized state.

### Implications for the properties of FeMo cofactor

**Figure 4:**
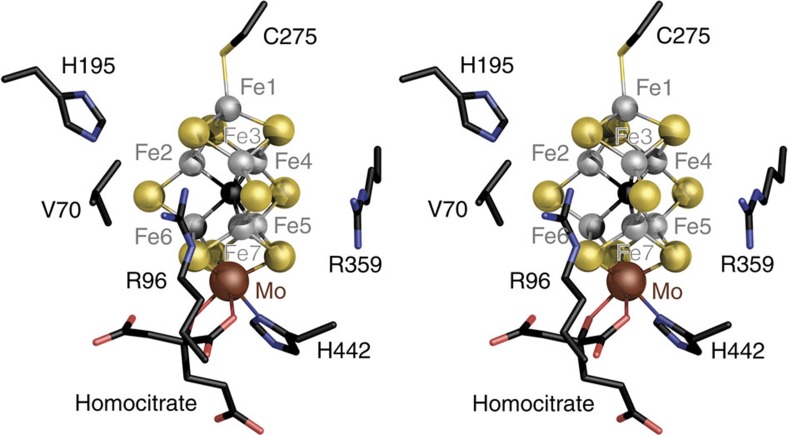
The protein environment of FeMo cofactor in *A.vinelandii* MoFe protein. The cluster is embedded between the three domains of the NifD subunit, with two conserved arginine residues arranged around one edge of FeMoco. The positive electrostatic potential field of the charged arginines R96 and R359 plausibly stabilizes the asymmetric charge distribution observed in the SpReAD analysis.

FeMo cofactor is a symmetric entity with an intrinsic pseudo-*D3* symmetry, where only the molybdenum ion breaks the twofold symmetry. The magnetic g tensor of the *S*=3/2 FeMoco^N^ state does not reflect this symmetry, and this distortion can likely be at least partially attributed to the inhomogeneous electrostatic potential field induced by the protein matrix^18^. The data presented here show that this also influences the electron distribution within the FeMoco, with the apparently more reduced irons Fe1, Fe3 and Fe7 grouping along one of the cluster edges. In the structure of nitrogenase MoFe protein, two conserved arginine residues, R96 and R359, line the Fe1-3-7-Mo edge of FeMo cofactor, and their positive charges may very well stabilize the more reduced iron edge (Fig. 4). The environment of the protein thus tunes the magnetic and electronic properties of the metal centre to create an intrinsic asymmetry of potential functional relevance. In the CO-bound structure of FeMoco, the ligand binds as a μ^2^-bridging metal carbonyl to Fe2 and Fe6, replacing a sulfido ligand^25^. This position is opposite form the reduced cluster edge with Fe1, Fe3 and Fe7 (Fig. 4). Interestingly, this is well in line with the mechanistic proposal advocated by Hoffman *et al*. that hinges on the storage of electrons in the form of metal hydrides on the cluster surface^36^. They assume hydride binding on the cluster face distant from the reduced edge and postulate a required reductive elimination of H_2_ concomitant with N_2_ binding, in line with our finding that electrons are directed away from the hydride-binding sites already in the resting state.

### Discussion

For a complex system such as FeMo cofactor, the assignment of integer redox states should be an oversimplification. However, in the literature, three oxidation state models are primarily discussed to represent the electron distribution in the FeMoco resting state based on the available spectroscopic data: [6Fe^+2^:1Fe^+3^:Mo^+4^], [4Fe^+2^:3Fe^+3^:Mo^+4^] and [2Fe^+2^:5Fe^+3^:Mo^+4^] (refs 37, 38, 39). None of these oxidation state models are in agreement with the SpReAD data reported here. The recent reassignment of the oxidation state of the apical molybdenum ion changed this picture, as the Mo ion was identified as a spin-coupled Mo^+3^ species and therefore the first example for a Mo^+3^ in a biological system^19^,^40^. Interestingly, the Mo-based catalytic complexes by Schrock and Nishibayashi also utilize a highly reduced molybdenum species with the metal in a Mo^+3^ state as a central intermediate^20^,^21^. Based on a Mo^+3^ state in FeMoco^N^, and in order to be consistent with the *S*=3/2 resting spin state, the previously assumed iron oxidation state models that necessarily resulted from the assignment of Mo^+4^ must be reconsidered. A *d*^3^ orbital configuration for the molybdenum (+3 oxidation state) would thus translate into the following possible redox models for the irons in FeMoco: [5Fe^+2^:2Fe^+3^:Mo^+3^], [3Fe^+2^:4Fe^+3^:Mo^+3^] and [1Fe^+2^:6Fe^+3^:Mo^+3^]. The presence of three reduced sites (Fe1/3/7) that are virtually identically to the iron sites in the P-cluster, in combination with the remaining four irons (Fe2/4/5/6) in a ‘more oxidized' state, is only in agreement with one of these redox state models. This is the [3Fe^+2^:4Fe^+3^:Mo^+3^] configuration that corresponds to a total cluster charge of −1. The ‘more oxidized' state we observe for Fe2, Fe4, Fe5 and Fe6 could indeed be Fe^+3^, but it is also conceivable that one or two electrons are delocalized to yield mixed-valence states. Upon inspection of the individual scattering curves, we find the four ‘more oxidized' iron sites to be very similar. Fe5 exhibits a slightly smaller edge shift than Fe4 (Figs 2 and 3), but as this difference is noticeably smaller than that of both sites to the three assigned Fe^+2^ sites Fe1, Fe3 and Fe7, it could possibly reflect the presence of residue R359 close to Fe5 that favours a charge shift from Fe4 to Fe5 (Fig. 4).

Combined with the assignment of Mo as Mo^+3^, the present study provides a first comprehensive picture of the electron distribution within FeMo-cofactor and therefore an experimental basis for the formulation of a comprehensive electronic description of the cluster through theoretical approaches. Such a model will be measured by its ability to reproduce the available spectroscopy and functional data, and it may in turn help to suggest new experiments. Eventually, understanding nitrogenase implies a detailed mechanism that necessarily involves the precise structural and electronic description of the catalytic intermediates laid out by the mechanism of Thorneley and Lowe^41^,^42^. For the development of the SpReAD method, further studies will be required to judge whether the analysis can be used to quantify oxidation states solely based on the position and fine structure of individual iron edges and thus being able to resolve partially delocalized states. Importantly, given the structural accessibility of ligand-bound FeMoco, SpReAD can provide spatially resolved electronic information about non-resting states of the active site to complement structural and spectroscopic data.

## Methods

### Protein production and isolation

*Azotobacter vinelandii* was grown aerobically on Burke's medium with sucrose as a carbon source, as described previously^10^. Expression of the *nif* genes was derepressed by nitrogen depletion, leading to a characteristic diauxic growth. Nitrogenase activity was monitored by detection of acetylene reduction via gas chromatography, and cells were harvested at or near the activity peak of the enzyme during the logarithmic portion of the second growth phase. All following steps were carried out under strict exclusion of dioxygen using modified Schlenk techniques and inert gas chambers.

### Crystallization and data collection

MoFe protein was crystallized in an anoxic chamber at less than 5 p.p.m. O_2_, using the sitting drop vapour diffusion technique at 291 K. Four microlitres of protein solution with a concentration of 80 mg ml^–1^ were mixed with 3 μl of reservoir solution containing 0.55 M NaCl, 16.5% (v/v) polyethylene glycol 6,000, 12.5% (v/v) methylpentane diol, 1.5% (v/v) xylitol, 0.2 M imidazole/malate buffer at pH 8.0, 0.55 mM spermine and 0.1 mM Zwittergent 3–14 (Hampton Research). Crystals were transferred to harvesting buffers with stepwise increase of methylpentane diol concentrations up to 19% (v/v), and flash-frozen in liquid nitrogen. Diffraction data were collected on beam line X06SA at the Swiss Light Source (Paul-Scherrer-Institut, Villigen, CH) and beam line 12-2 at the Stanford Synchrotron Radiation Laboratory, using Dectris Pilatus 6M detectors. Crystals of nitrogenase MoFe protein belonged to the monoclinic space group *P*2_1_, with one NifD_2_K_2_ heterotetramer per asymmetric unit and unit cell dimensions of *a*=80.9 Å, *b*=130.8 Å, *c*=107.0 Å, *β*=110.6°. Seventeen oscillation data sets over 200° in steps of 0.5° were collected at different spots on a single crystal, largely in steps of 2 eV along the iron K-edge at 7,121 eV and individually integrated and merged using XDS^43^ and SCALA (Supplementary Table 1)^44^. No deterioration of data quality was observed during the measurement. For every crystal used for data collection, electron density maps were calculated and inspected in order to confirm the reduced state of the enzyme from the conformation of atoms Fe5 and Fe6 of the two the P-clusters per asymmetric unit. These undergo a reversible positional shift between the all-ferrous P^N^ and the two-electron oxidized P^Ox^ state that is straightforward to discern^35^. The data sets were then merged into a single MTZ file using CAD and scaled with SCALEIT^45^. In parallel, the model of *A. vinelandii* MoFe protein (PDB ID 3U7Q) was refined against the data set collected at the lowest X-ray energy using REFMAC^46^ and used as a source for phase information during the subsequent structure factor calculation (Supplementary Fig. 3).

### SpReAD analysis

Anomalous scattering describes the breakdown of Friedel's law^32^, the intrinsic inversion symmetry of diffraction (*F*(**S**)=*F*(−**S**)), in the proximity of an X-ray absorption edge. Anomalous scattering is routinely used to solve the crystallographic phase problem in the *ab initio* structure determination of proteins^33^, and its magnitude across an edge is proportional to the absorption of X-rays. The atomic scattering factor *f*(*λ*) shows a dependence on X-ray energies that is proportional to their absorption by atoms of a given element. According to *f*(*λ*)=*f* ^0^+Δ*f* ′+*i*Δ*f* ″, this leads to a dispersive (real) and an anomalous (imaginary) absorption correction of the atomic scattering factor *f* ^0^ that directly relates to the shape of an X-ray absorption spectrum. Because of the spatial resolution of a diffraction data set, the properties Δ*f* ′ and Δ*f* ″ can be refined for each individual atom, yielding dispersive (Δ_disp_) and anomalous (Δ_ano_) differences for every single structure factor **S**=(*h k l*). To obtain **S**, the measured anomalous differences at each energy are used as amplitudes, while phase angles are derived from a refined structural model.


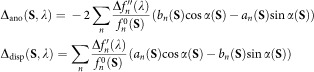


Here, 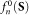 is the atomic scattering factor (disregarding anomalous scattering) for the *n*th atom of the scatterer in question, *λ* is the X-ray wavelength, *α* is the phase angle of a given reflection and *a*_*n*_(**S**) and *b*_*n*_(**S**) are the real and imaginary components of the atomic scattering factor, respectively^31^. The above properties were refined for all anomalous scatterers in the asymmetric unit of the MoFe protein crystals. Hereby, C, N, O, S and Ca did not show edge features, so that the sum of all atoms of each type was used as a single term, whereas the 30 iron atoms in question were refined individually.

A structural model of MoFe protein was refined against the data set collected at 7,095 eV and inspection of the electron density maps confirmed the P-cluster to be entirely in the P^N^ state. For each iron atom in FeMo cofactor and in the P-cluster, an individual refinement of the anomalous *f* ″ and the dispersive *f* ′ contribution of anomalous scattering was then carried out as described previously^31^. The asymmetric unit of the *P*2_1_ crystals of *A. vinelandii* MoFe protein contained one heterotetramer with two copies each of P-cluster (8 Fe) and FeMo cofactor (7 Fe). The two instances of each cluster were inspected separately for comparison and yielded virtually identical results. We considered whether the relative orientation of the metal clusters to the polarized synchrotron beam should have a significant influence on individual absorption edges, but found such effects to be minor in our SpReAD data (Supplementary Fig. 1). Thirty individual Fe atoms were included in the refinement, as well as the other anomalous scatterers present in the crystal, Mo, S and Ca. The absorption behaviour of these elements is featureless around the iron K-edge, but they contribute to the total anomalous scattering and were thus included, albeit only as the sum of all atoms of the given type.

## Additional information

**How to cite this article:** Spatzal, T. *et al*. Nitrogenase FeMoco investigated by spatially resolved anomalous dispersion refinement. *Nat. Commun.* 7:10902 doi: 10.1038/ncomms10902 (2016).

## Supplementary Material

Supplementary Information Supplementary Figures 1-3.

## Figures and Tables

**Table 1:** Data collection and refinement statistics.

*Data collection*																	
Space group	*P*2_1_																
*Cell dimensions*																	
*a*, *b*, *c* (Å)	80.9, 130.8, 107.0																
*α, β, γ* (°)	90.0, 110.6, 90.0																
Resolution (Å)	50.0–2.10 (2.22–2.10)*																
*Energy (eV)*	**7,103**	**7,108**	**7,110**	**7,112**	**7,114**	**7,116**	**7,118**	**7,120**	**7,122**	**7,124**	**7,126**	**7,128**	**7,130**	**7,132**	**7,134**	**7,136**	**7,141**
*R*_merge_	0.130 (0.275)	0.120 (0.251)	0.116 (0.236)	0.112 (0.222)	0.106 (0.208)	0.102 (0.195)	0.099 (0.181)	0.096 (0.171)	0.093 (0.163)	0.091 (0.157)	0.088 (0.148)	0.087 (0.143)	0.084 (0.133)	0.082 (0.125)	0.080 (0.122)	0.140 (0.282)	0.143 (0.295)
*I*/σ*I*	6.8 (4.6)	7.2 (4.9)	7.3 (5.1)	7.6 (5.2)	8.2 (5.6)	8.4 (5.9)	8.8 (6.2)	8.8 (6.5)	9.2 (6.7)	9.2 (6.9)	9.6 (7.3)	10.0 (7.1)	10.2 (7.8)	10.5 (8.2)	10.9 (8.5)	6.2 (4.1)	6.4 (4.1)
Unique reflections	115,534 (16,704)	115,667 (16,731)	115,861 (16,771)	116,006 (16,790)	116,035 (16,799)	116,194 (16,827)	116,192 (16,832)	116,335 (16,850)	116,496 (16,881)	116,523 (16,885)	116,638 (16,914)	116,609 (16,919)	116,783 (16,943)	116,761 (16,948)	116,906 (16,968)	117,562 (17,001)	117,326 (17,021)
Completeness (%)	96.3 (95.7)	96.3 (95.7)	96.3 (95.7)	96.3 (95.7)	96.3 (95.8)	96.3 (95.8)	96.3 (95.8)	96.3 (95.8)	96.3 (95.8)	96.3 (95.8)	96.3 (95.8)	96.3 (95.9)	96.3 (95.8)	96.3 (95.8)	96.2 (95.9)	96.3 (95.5)	96.3 (95.8)
Multiplicity	3.5	3.5	3.5	3.5	3.5	3.5	3.5	3.5	3.5	3.5	3.5	3.5	3.5	3.5	3.4	3.5	3.5
Anom. Compl. (%)	87.1 (86.1)	87.0 (86.2)	87.1 (86.2)	87.1 (86.3)	87.0 (86.3)	87.0 (86.2)	86.9 (86.3)	86.8 (86.3)	86.7 (86.3)	86.7 (86.4)	86.6 (86.4)	86.5 (86.5)	86.4 (86.6)	86.2 (86.6)	86.3 (86.6)	87.4 (85.9)	87.1 (86.4)
Scale factor	1.000 (1.000)	1.002 (1.011)	0.998 (1.008)	1.000 (1.007)	1.001 (1.011)	1.003 (1.014)	1.001 (1.013)	0.998 (1.015)	1.001 (1.016)	0.999 (1.016)	1.002 (1.015)	1.001 (1.015)	1.000 (1.016)	1.001 (1.014)	1.001 (1.012)	0.998 (1.018)	1.003 (0.992)
wt. *R* for scaling	0.000	0.142	0.139	0.137	0.132	0.132	0.128	0.128	0.126	0.128	0.126	0.126	0.127	0.122	0.119	0.158	0.161
Δ_ano_/*σ*(Δ_ano_)	0.68	0.69	0.68	0.70	0.69	0.70	0.71	0.71	0.75	0.76	0.77	0.80	0.80	0.82	0.83	0.76	0.73
																	
*Refinement*																	
*R*_work_	0.1902																
*R*_free_	0.2264																
*R.m.s. deviations*																	
Bond lengths (Å)	0.025																
Bond angles (°)	2.332																

Abbreviations: Anom. Compl., anomalous completeness; r.m.s., root-mean-squared.

^*^Highest resolution shell parenthesis. Bold entries represent the X-ray energies for the individual data sets (in unit of electron volts), which was the main parameter varied in the experiment.
