# Supplementary material for: Nitrogenase FeMoco investigated by spatially resolved anomalous dispersion refinement
Source: Nat Commun. 2016 Mar 14;7:10902. doi: 10.1038/ncomms10902 (PMC4793075; doi:10.1038/ncomms10902)
Supplement: Supplementary Information — Supplementary Figures 1-3. [file ncomms10902-s1.pdf]

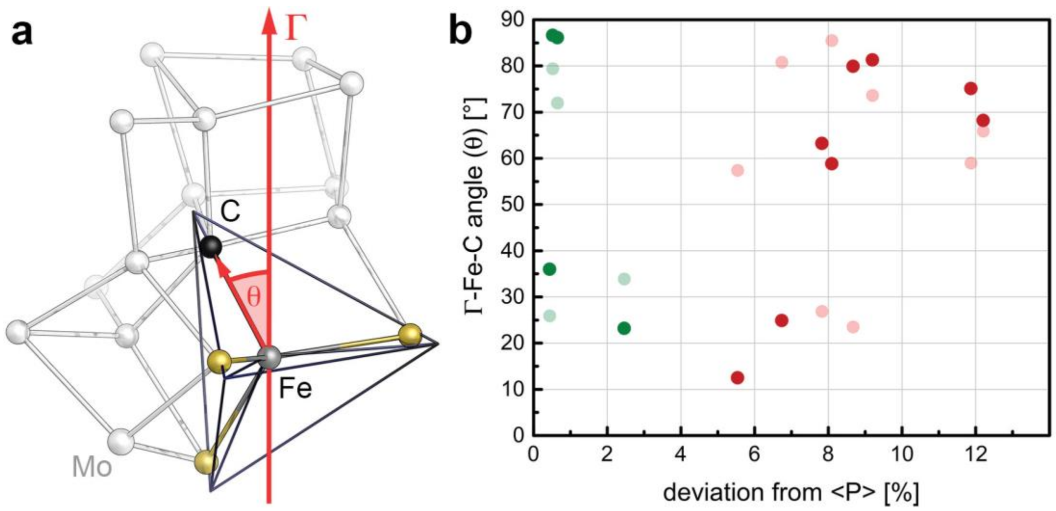

Supplementary Figure 1: An estimate of polarization effects on the SpReAD analysis. For the synchrotron beam lines used in this work, the polarization vector **Γ** of X-ray radiation was parallel to the rotation axis of the crystal during data collection. It was thus possible to define an angle θ to relate the direction of **Γ** to the direction of the Fe–C bond for each of the 6 Fe sites that form the trigonal prism of FeMoco. As their chemical environment is identical, a strong influence of polarization effects was expected to manifest in a correlation between θ and the observed position of the absorption edge. **A**) θ was determined for the two copies of FeMoco per MoFe-heterotetramer as well as in the additional $P2_1$ symmetry related metal centers as the angle between each Fe-C bond and the polarization vector **Γ**. **B**) A scatter plot relating the relative deviations of the edge position from the P-cluster average (Fig. 2) to the angles θ (Fe3,7 are shown in green and Fe2,4,5,6 in red; lighter colors indicate the symmetry mate related by a crystallographic $P2_1$ operation). The plot should indicate correlation if the edge position depended on θ, but only yields R_1 = 0.19 and R_2 = 0.09 for the two copies of FeMo cofactor respectively. Evidently, polarization effects are minor in the present experiment.

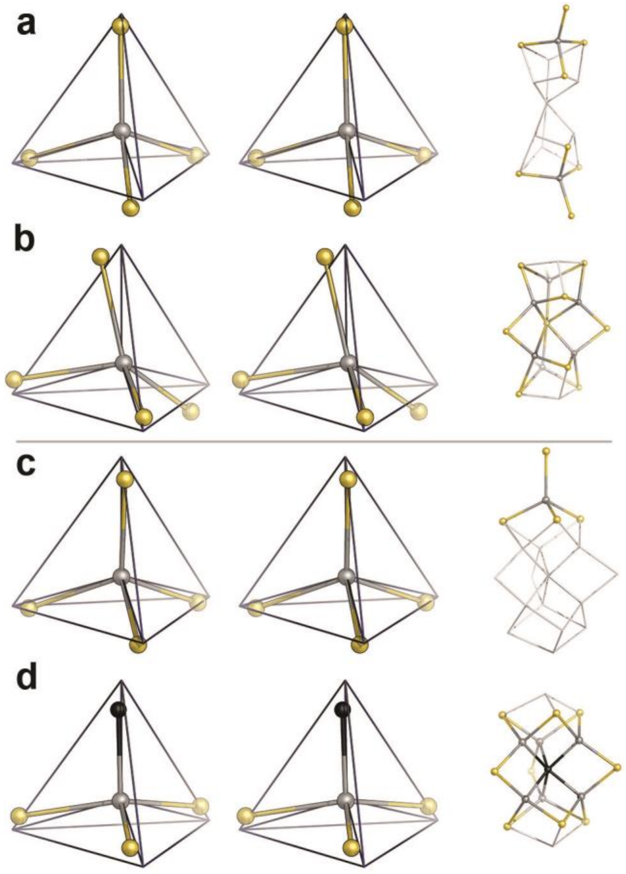

Supplementary Figure 2: Ligand field geometries for the iron sites in MoFe protein. **A**) In P-cluster in the P^N state, Fe3 and Fe7 show least distortion from tetrahedral geometry. **B**) The remaining four Fe ions of P-cluster coordinate the central sulfur ligand and are thus slightly distorted. **C**) Fe1, the apical iron of FeMoco, has four sulfido ligands, resulting in a minimally distorted tetrahedral geometry. **D**) The geometrically identical Fe2-Fe7 of FeMo cofactor form a trigonal prism with the interstitial carbide in its center. The tetrahedral ligand field of these atoms is distorted, with the iron atom shifted closer to the plane formed by the three sulfide ions and a shortened bond length (2.0 Å) to the carbide ligand.

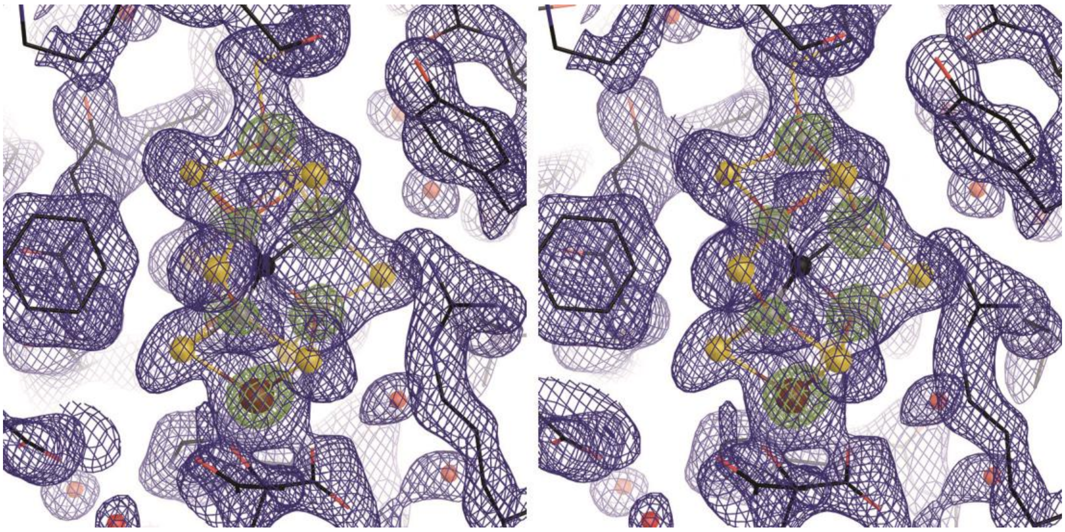

Supplementary Figure 3: Electron density maps for nitrogenase, recorded at the Fe K-edge (7122 eV). The stereo image shows the active site FeMo cofactor within a $2F_o–F_c$ electron density map contoured at the 1σ level (blue). An anomalous difference electron density map contoured at the 3σ level is shown green.
